# Supplementary material for: Meta-Analysis and Gene Set Enrichment Relative to ER Status Reveal Elevated Activity of MYC and E2F in the “Basal” Breast Cancer Subgroup
Source: PLoS One. 2009 Mar 9;4(3):e4710. doi: 10.1371/journal.pone.0004710 (PMC2650420; doi:10.1371/journal.pone.0004710)
Supplement: Dataset S1 — GSEA results from first screen. The file contains a zipped archive of the HTML results of the second GSEA screen. Reports for enriched or depleted datasets may be accessed from the “index.html” link within the my_analysis.GseaPreranked.1219975950625.rpt.zip ZIP archive. Enrichment plots are available for each of the 50 most enriched and depleted gene sets. (13.32 MB ZIP) [file pone.0004710.s001.zip › my_analysis.GseaPreranked.1219975950625.rpt.zip!//CHR16P12.html]

Details for gene set CHR16P12[GSEA]

|  || Dataset | zSco.ERneg.to.ERpos\_collapsed |
| Phenotype | NoPhenotypeAvailable |
| Upregulated in class | na\_neg |
| GeneSet | CHR16P12 |
| Enrichment Score (ES) | -0.47380248 |
| Normalized Enrichment Score (NES) | -1.9918824 |
| Nominal p-value | 0.0 |
| FDR q-value | 0.00938164 |
| FWER p-Value | 0.205 |
Table: GSEA Results Summary

  

Fig 1: Enrichment plot: CHR16P12      
 Profile of the Running ES Score & Positions of GeneSet Members on the Rank Ordered List

  

| PROBE | GENE SYMBOL | GENE\_TITLE | RANK IN GENE LIST | RANK METRIC SCORE | RUNNING ES | CORE ENRICHMENT || 1 | GPRC5B |  |  | 103 | 5.845 | 0.0477 | No |
| 2 | XPO6 |  |  | 2884 | 1.993 | -0.1430 | No |
| 3 | PRKCB1 |  |  | 2915 | 1.980 | -0.1265 | No |
| 4 | SCNN1G |  |  | 3164 | 1.859 | -0.1276 | No |
| 5 | TCEB2 |  |  | 3437 | 1.735 | -0.1316 | No |
| 6 | SLC5A2 |  |  | 3843 | 1.573 | -0.1473 | No |
| 7 | LOC94431 |  |  | 3932 | 1.540 | -0.1393 | No |
| 8 | METTL9 |  |  | 4070 | 1.485 | -0.1355 | No |
| 9 | RRN3 |  |  | 4108 | 1.469 | -0.1244 | No |
| 10 | DNAH3 |  |  | 4349 | 1.372 | -0.1295 | No |
| 11 | DCTN5 |  |  | 4414 | 1.349 | -0.1215 | No |
| 12 | SMG1 |  |  | 4882 | 1.194 | -0.1454 | No |
| 13 | ARHGAP17 |  |  | 4933 | 1.177 | -0.1380 | No |
| 14 | IGSF6 |  |  | 4987 | 1.160 | -0.1310 | No |
| 15 | ZP2 |  |  | 5282 | 1.055 | -0.1432 | No |
| 16 | ERN2 |  |  | 5558 | 0.956 | -0.1548 | No |
| 17 | RPS15A |  |  | 5980 | 0.821 | -0.1788 | No |
| 18 | LOC440345 |  |  | 6108 | 0.780 | -0.1810 | No |
| 19 | LOC400506 |  |  | 6863 | 0.530 | -0.2328 | No |
| 20 | AQP8 |  |  | 7040 | 0.472 | -0.2416 | No |
| 21 | NOMO2 |  |  | 7364 | 0.361 | -0.2625 | No |
| 22 | SCNN1B |  |  | 7413 | 0.342 | -0.2629 | No |
| 23 | RBBP6 |  |  | 8154 | 0.055 | -0.3182 | No |
| 24 | GP2 |  |  | 8456 | -0.058 | -0.3403 | No |
| 25 | ACSM1 |  |  | 8567 | -0.108 | -0.3476 | No |
| 26 | ATP2A1 |  |  | 8578 | -0.116 | -0.3472 | No |
| 27 | PALB2 |  |  | 8670 | -0.158 | -0.3526 | No |
| 28 | UQCRC2 |  |  | 8708 | -0.175 | -0.3537 | No |
| 29 | FLJ20581 |  |  | 8971 | -0.301 | -0.3706 | No |
| 30 | LOC23117 |  |  | 9137 | -0.378 | -0.3795 | No |
| 31 | LOC63928 |  |  | 9327 | -0.472 | -0.3893 | No |
| 32 | CP110 |  |  | 9386 | -0.500 | -0.3889 | No |
| 33 | HS3ST2 |  |  | 9761 | -0.701 | -0.4104 | No |
| 34 | JMJD5 |  |  | 9866 | -0.764 | -0.4110 | No |
| 35 | ARL6IP1 |  |  | 9916 | -0.793 | -0.4072 | No |
| 36 | NDUFAB1 |  |  | 10064 | -0.886 | -0.4099 | No |
| 37 | LOC81691 |  |  | 10378 | -1.081 | -0.4232 | No |
| 38 | COQ7 |  |  | 10728 | -1.333 | -0.4369 | No |
| 39 | UBPH |  |  | 10777 | -1.371 | -0.4275 | No |
| 40 | IQCK |  |  | 11056 | -1.622 | -0.4331 | No |
| 41 | RABEP2 |  |  | 11079 | -1.647 | -0.4192 | No |
| 42 | PPP4C |  |  | 11470 | -2.005 | -0.4295 | No |
| 43 | PYCARD |  |  | 11774 | -2.297 | -0.4306 | No |
| 44 | SULT1A1 |  |  | 12348 | -3.019 | -0.4452 | Yes |
| 45 | EXOD1 |  |  | 12369 | -3.052 | -0.4177 | Yes |
| 46 | CDIPT |  |  | 12464 | -3.206 | -0.3944 | Yes |
| 47 | SULT1A2 |  |  | 12505 | -3.268 | -0.3665 | Yes |
| 48 | HIRIP3 |  |  | 12659 | -3.540 | -0.3444 | Yes |
| 49 | MIR16 |  |  | 12715 | -3.646 | -0.3140 | Yes |
| 50 | POLR3E |  |  | 12770 | -3.791 | -0.2821 | Yes |
| 51 | LCMT1 |  |  | 12813 | -3.917 | -0.2481 | Yes |
| 52 | GTF3C1 |  |  | 12854 | -4.018 | -0.2131 | Yes |
| 53 | CLN3 |  |  | 12965 | -4.376 | -0.1799 | Yes |
| 54 | MGC16824 |  |  | 13044 | -4.624 | -0.1419 | Yes |
| 55 | SYT17 |  |  | 13173 | -5.194 | -0.1023 | Yes |
| 56 | COG7 |  |  | 13225 | -5.622 | -0.0528 | Yes |
| 57 | KIAA0556 |  |  | 13280 | -6.318 | 0.0030 | Yes |
Table: GSEA details [plain text format]

  

Fig 2: CHR16P12: Random ES distribution      
 Gene set null distribution of ES for **CHR16P12**
